# Surviving All Odds: A Unique Case of Multiple Congenital Unruptured Sinus of Valsalva Aneurysms Involving Both Left and Right Coronary Sinuses with Biventricular Dysfunction and Heart Block

**DOI:** 10.1155/2016/4654031

**Published:** 2016-11-02

**Authors:** Aniketh Vijay B, Vikrant Vijan, Navin Mathew

**Affiliations:** Amrita Institute of Medical Sciences (AIMS), Amrita Vishwa Vidyapeetham University, Kochi, India

## Abstract

Aneurysms of the sinus of Valsalva are very uncommon, with an incidence ranging from 0.1 to 3.5% of all congenital heart defects. Very few cases have been reported in the literature that presented with involvement of two or more sinuses. We report a case of 27-year-old male with a history of exertional breathlessness of one-month duration. After complete evaluation using transesophageal echocardiography (TEE) and multiple detector computed tomography (MDCT) scanning, the patient was diagnosed to have large congenital unruptured sinus of Valsalva aneurysms involving both left and right coronary sinuses with extension into the interventricular septum. The patient also displayed second-degree heart block (Mobitz type 2) and biventricular dysfunction. The patient was managed successfully. We present the case with an aim to highlight the management challenges including intraoperative and postoperative complications that are associated with unruptured sinus of Valsalva aneurysms of ≥2 sinuses.

## 1. Introduction

Aneurysm of sinus of Valsalva is a rare congenital anomaly [1]. Further, the aneurysms of the sinus of Valsalva rarely present with symptoms unless rupture occurs [2]. We describe a symptomatic case of multiple unruptured aneurysms of sinus of Valsalva, involving right and left aortic sinuses, with extensive dissection into the interventricular septum.

## 2. Case Report

A 27-year-old male with moderate build (body mass index: 20 kg/m^2^) presented to our outpatient department with a history of exertional dyspnea (class I) of one-month duration with no history of associated chest pain or orthopnea. He was apparently normal otherwise. Elsewhere, he was evaluated by a cardiologist. His echocardiogram had revealed dilated aorta, left ventricular dysfunction, and pulmonary hypertension. The patient was referred to our hospital for complete evaluation and management.

**Figure 1:**
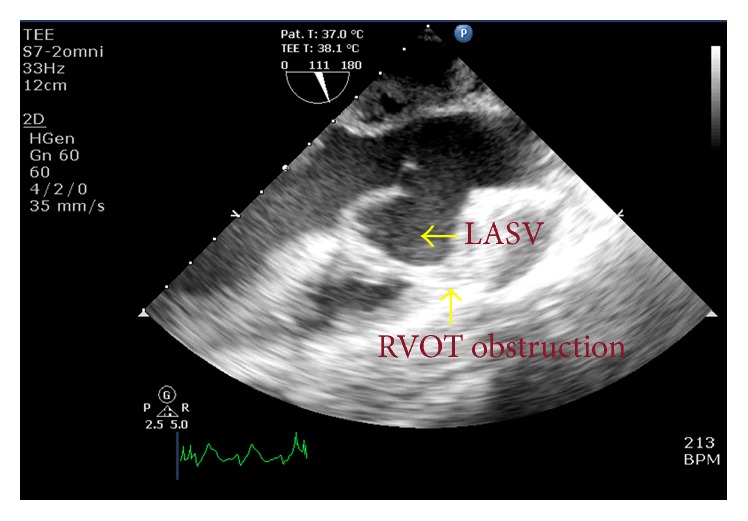
Transesophageal echocardiography^*∗*^ revealed left sinus of Valsalva aneurysm with thrombus within it causing RVOT obstruction. ^*∗*^Aortic valve long-axis view at midesophageal level.

**Figure 2:**
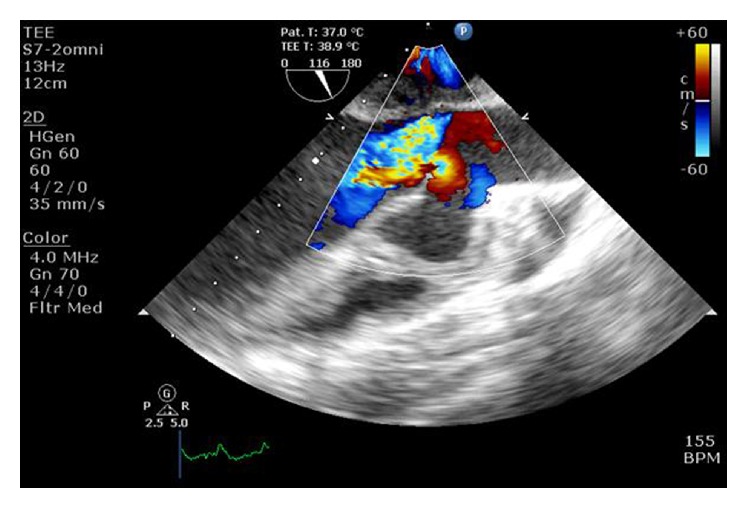
Transesophageal echocardiography^*∗*^ with color Doppler revealed mild-to-moderate aortic regurgitation due to the prolapse of the left coronary cusp into the left aneurysm of sinus of Valsalva. ^*∗*^Aortic valve long-axis view at midesophageal level.

**Figure 3:**
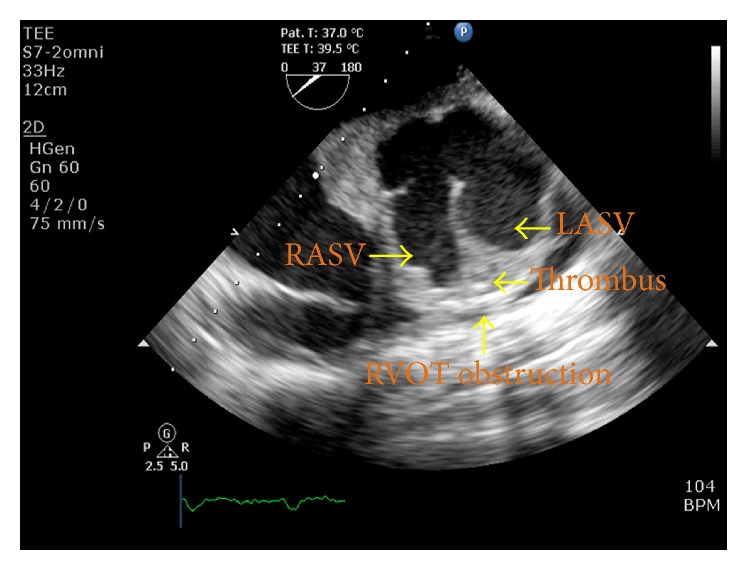
Transesophageal echocardiography^*∗*^ revealed left aneurysm of sinus of Valsalva (LASV) and right aneurysm of sinus of Valsalva (RASV) with thrombus within it, causing RVOT obstruction. ^*∗*^Aortic valve short-axis view at midesophageal level.

**Figure 4:**
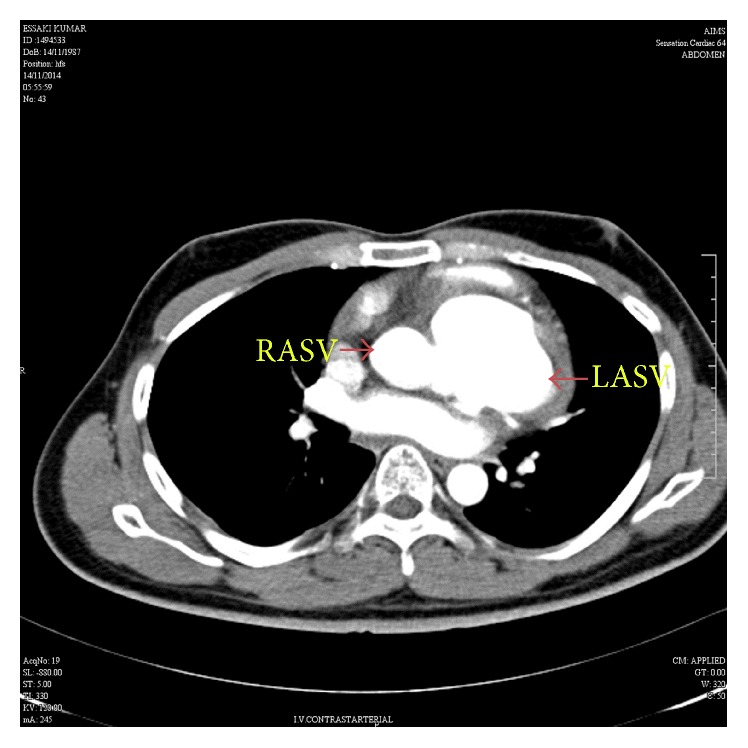
MDCT aortogram suggested left aneurysm of sinus of Valsalva (LASV) and right aneurysm of sinus of Valsalva (RASV) with extension into the interventricular septum.

**Figure 5:**
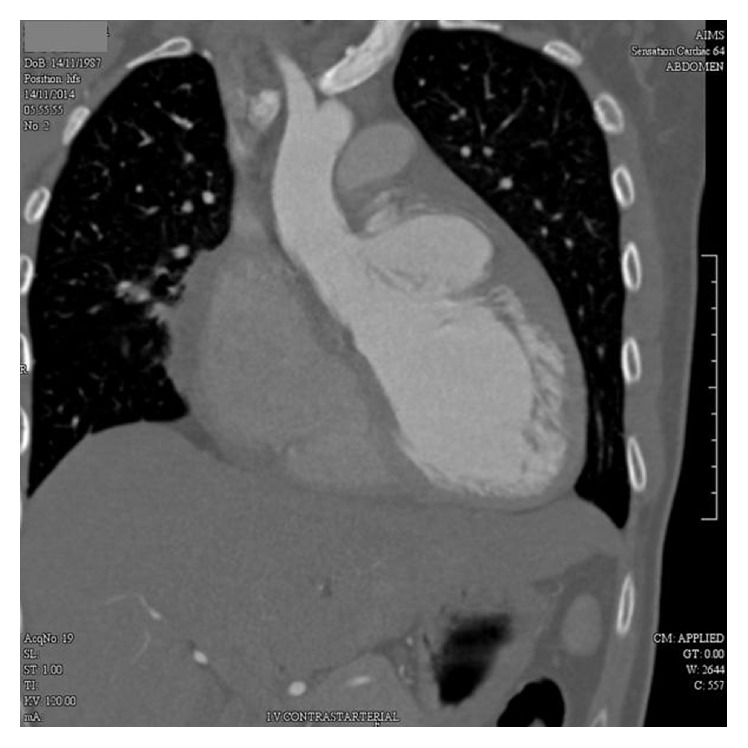
MDCT chest coronal section confirmed left and right sinus of Valsalva aneurysms.

**Figure 6:**
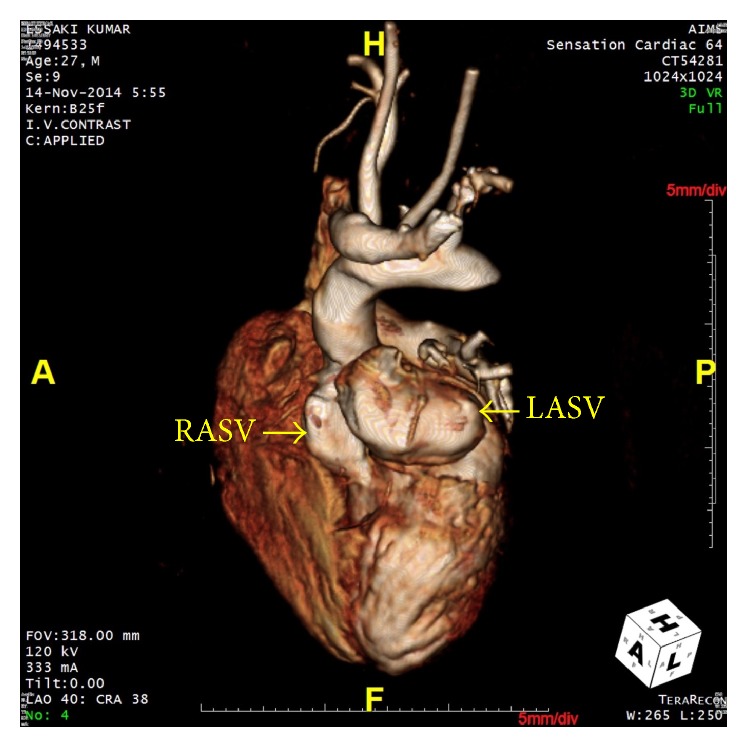
The 3D reconstructed image with MDCT aortogram revealed left aneurysm of sinus of Valsalva (LASV; size: 5.4 × 5.7 cm) and right aneurysm of sinus of Valsalva (RASV; size: 4.6 × 2.7 cm) with extension into the interventricular septum; left coronary artery arising from the roof of the left diverticulum.

**Figure 7:**
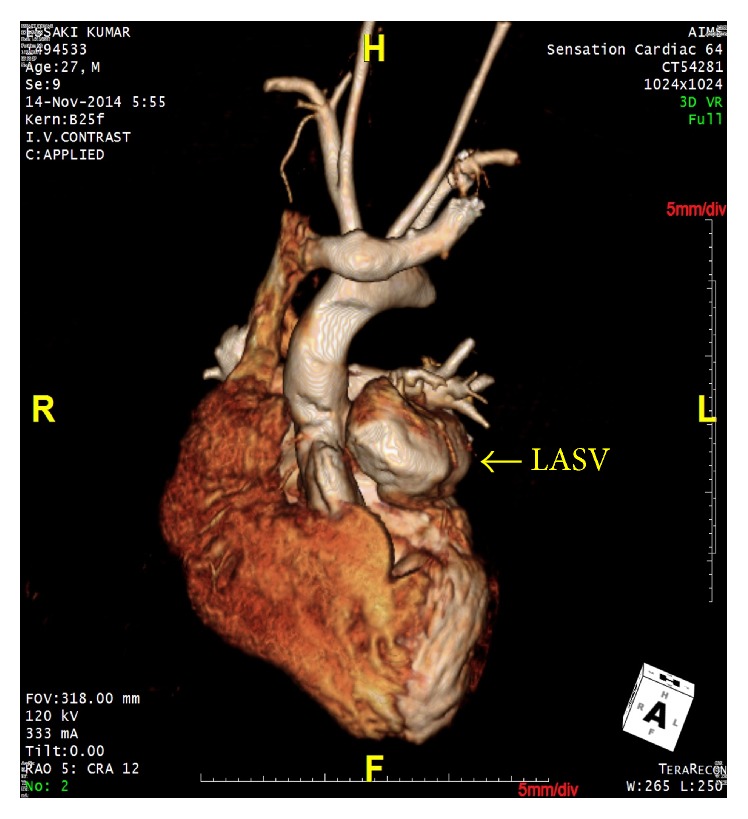
A 3D reconstructed MDCT chest coronal section image indicating left aneurysm of sinus of Valsalva (LASV).

At the time of presentation, the patient had a pulse rate of 80 bpm and the blood pressure of 110/80 mmHg. Clinical examination revealed left ventricular apex, physiological splitting of S2, and ejection systolic murmur of grade 3/6 in the left upper sternal border. A 12-lead electrocardiogram was performed, which showed right bundle branch block with first-degree heart block. Chest X-ray revealed cardiomegaly. Subsequent examination with 2D transthoracic echocardiography (TTE) revealed diverticular formation from both left and right aortic sinuses. Transesophageal echocardiography (TEE) further confirmed the findings of diverticular formation from both left and right aortic sinuses (Figure 1). It also revealed that the diverticulum from the right sinus extended into the interventricular septum. Further, aortic regurgitation of mild-to-moderate severity was noted due to the prolapse of left cusp into the left aneurysm of sinus of Valsalva (Figure 2). Biventricular dysfunction was present and aneurysm arising from the left aortic sinus contained a large layered thrombus, causing right ventricular outflow tract (RVOT) obstruction (RVOT gradient: 30 mmHg) (Figure 3). For further confirmation, multiple detector computed tomography (MDCT) scanning was performed (Figures 4 and 5), which revealed a diverticulum on both right and left coronary sinuses: a bilobed diverticulum from the left sinus with a maximum diameter of 5.4 × 5.7 cm was directed inferiorly and to left side (Figure 6), while a 4.6 × 2.7 cm right diverticulum from the right sinus was bulging into the interventricular septum and was directed anteroinferiorly (Figure 6). Further, the right coronary artery was seen arising on the side of the right diverticulum, while left coronary artery was seen arising from the roof of left diverticulum (Figure 7). Holter monitoring revealed an intermittent second-degree atrioventricular block (Mobitz type 2). Subsequently, all the acquired causes of sinus of Valsalva aneurysms were ruled out by necessary investigations.

Patient was planned for Bentall procedure and permanent pacemaker implantation. Intraoperative aortotomy was performed, following which there was dissection around root, leading to injury in the ostium segment of right coronary artery (RCA) and narrowing of the ostium segment of left main coronary artery. Hence, CABG was decided. The aortic root was replaced with a 23 mm St. Jude medical mechanical valve conduit. Later, CABG was performed for left anterior descending (LAD) artery, obtuse marginal artery, and distal RCA from reversed saphenous vein grafts. Postoperatively, the patient developed congestive heart failure. Echocardiogram displayed a large pericardial effusion which was drained by left lateral thoracotomy. After three days, a permanent pacemaker (St. Jude Medical) was implanted for second-degree atrioventricular block. Patient's condition improved subsequently and he was discharged after 2 days after an uneventful hospital stay. At follow-up after about 8 months, the patient remained asymptomatic and was leading a regular healthy life. Repeated TTE revealed a significant improvement in the biventricular function and significant reduction in the RVOT gradient.

## 3. Discussion

Sinus of Valsalva aneurysms is extremely rare, accounting for about 0.1% to 3.5% of all the congenital heart defects [1]. Among the sinuses usually involved, it has been estimated that involvement of right coronary sinus is the most frequent, followed by noncoronary sinus, while left coronary sinus involvement is the rarest of all [3]. Further, the cases of multiple aneurysms of sinus of Valsalva are very rare. Here, we present a case of multiple aneurysms of sinus of Valsalva with involvement of left coronary sinus along with right coronary sinus.

In general, patients with unruptured aneurysm of sinus of Valsalva are usually asymptomatic until a rupture occurs into one of the heart chambers, leading to overt heart failure. Such patients with unruptured sinus of Valsalva aneurysm are usually diagnosed incidentally during routine echocardiography. However, there have been very few reports which describe a presentation of symptomatic patients with unruptured aneurysm of sinus of Valsalva [2]. Conversely, the patient in the present case presented with class I exertional dyspnea with no history of associated chest pain. He also exhibited pulmonary hypertension, biventricular dysfunction, second-degree heart block, mild-to-moderate aortic regurgitation, right bundle branch block, thrombus within an aneurysmal sac, and RVOT obstruction.

In general, two-dimensional and Doppler echocardiography is adequate to diagnose unruptured sinus of Valsalva aneurysms [2]. In addition to this, we used MDCT scanning to further confirm the diagnosis and to characterize the precise size of the aneurysms in three dimensions. We ruled out all the acquired causes of sinus of Valsalva aneurysm (i.e., atherosclerotic, infective, medial cystic necrosis, connective tissue disorders, or trauma) [4] by doing the necessary investigations in the present case.

The management of unruptured aneurysm of sinus of Valsalva generally involves surgical repair [2]. In the present case, the patient underwent Bentall procedure. However, coronary artery bypass grafting (CABG) was performed as a result of an intraoperative complication including injury in the ostium RCA, narrowing of ostium left main coronary artery, and pericardial effusion, which were managed successfully. Subsequently, a permanent pacemaker was implanted. After procedure, the condition of the patient improved significantly and he remained asymptomatic on 8-month follow-up.

## 4. Conclusion

We suggest fellow interventional cardiologists that appropriate prophylactic surgical treatment of the aneurysm may help in preventing intraoperative or postoperative complications.

## References

[1] Post M. C., Braam R. L., Groenemeijer B. E., Nicastia D., Rensing B. J., Schepens M. A. (2010). Rupture of right coronary sinus of Valsalva aneurysm into right ventricle. *Netherlands Heart Journal*.

[2] Thankachen R., Gnanamuthu R., Doshi H., Shukla V., Korula R. J. (2003). Unruptured aneurysm of the sinus of valsalva: presenting with right ventricular outflow obstruction. *Texas Heart Institute Journal*.

[3] Munjewar C. B., Agrawal R. D., Sharma S. (2014). An unusual case of rupture of left sinus of valsalva aneurysm into main pulmonary artery. *Annals of Pediatric Cardiology*.

[4] Zhang J., Liu Y., Liu L., Deng Y. (2016). An extracardiac unruptured right sinus of valsalva aneurysm complicated with atherothrombosis. *Echo Research and Practice*.

